# Bibliometric mapping of mesenchymal stem cell therapy for bone regeneration from 2013 to 2023

**DOI:** 10.3389/fmed.2024.1484097

**Published:** 2025-01-06

**Authors:** Qianqian Chen, Yiqi Su, Zhen Yang, Qiyuan Lin, Yan Ke, Dan Xing, Hui Li

**Affiliations:** ^1^Third Affiliated Hospital of Zhejiang Chinese Medical University, Hangzhou, China; ^2^Arthritis Clinic & Research Center, Zhejiang Chinese Medical University, Hangzhou, China; ^3^Arthritis Clinic & Research Center, Peking University People’s Hospital, Peking University, Beijing, China; ^4^Arthritis Institute, Peking University, Beijing, China

**Keywords:** visualization research, mesenchymal stem cell, cell therapy, bone regeneration, CiteSpace, VOSviewer

## Abstract

Mesenchymal stem cells (MSCs) have shown significant potential in bone regeneration and regenerative medicine in recent years. With the advancement of tissue engineering, MSCs have been increasingly applied in bone repair and regeneration, and their clinical application potential has grown through interdisciplinary approaches involving biomaterials and genetic engineering. However, there is a lack of systematic reviews summarizing their applications in bone regeneration. To address this gap, we analyzed the latest research on MSCs for bone regeneration published from 2013 to 2023. Using the Web of Science Core Collection, we conducted a literature search in December 2024 and employed bibliometric tools like CiteSpace and VOSviewer for a comprehensive analysis of the key research trends. Our findings focus on the development of cell engineering, highlighting the advantages, limitations, and future prospects of MSC applications in bone regeneration. These insights aim to enhance understanding of MSC-based bone regeneration, inspire new research directions, and facilitate the clinical translation of MSC research.

## 1 Introduction

Bones have a unique scarless regenerative capacity, allowing them to completely repair damaged areas and restore their normal shape and function. Unlike most tissues, bone tissue does not form scars after injury but instead undergoes a complete regeneration process through a series of steps (1, 2). This process primarily relies on two pathways: intramembranous ossification and endochondral ossification. These two mechanisms play a crucial role in bone regeneration, jointly facilitating the restoration and repair of bone tissue (3–5).

Mesenchymal stem cells (MSCs) are multipotent stem cells found primarily in bone marrow, periosteum, and endosteum. These cells can differentiate into osteoblasts (bone-forming cells) and chondrocytes (cartilage-forming cells), playing a key role in fracture repair (6–10). MSCs not only promote bone healing by directly forming bone and cartilage but also influence the healing process indirectly through the secretion of cytokines, regulation of angiogenesis, and modulation of inflammatory responses (11, 12). Therefore, MSCs hold great potential in the fields of bone regeneration and tissue engineering.

However, in conventional clinical trials for bone repair, stem cell therapy faces challenges such as significant cell loss post-transplantation, apoptosis, and poor targeting (13, 14). Consequently, it is crucial to target the delivery of MSCs to the site of bone injury or defect to optimize their regenerative effects. Currently, researchers have adopted various strategies to enhance MSC homing and transplantation, including the use of biomaterial scaffolds, growth factors, and cell surface modifications (15–19). These methods aim to improve MSC targeting and survival rates at the target site, thereby promoting tissue regeneration and repair. Despite significant breakthroughs in mesenchymal stem cell therapy for bone regeneration over the past decade, there is still a lack of systematic reviews on its application in this field.

Bibliometric analysis is a method that can quantitatively and qualitatively analyze authors, journals, research teams, sponsoring institutions, or countries, to describe the current state of research and predict trends in related fields (20). Therefore, this paper employs bibliometric analysis to conduct an in-depth examination of the relevant literature, exploring the current status, advantages, limitations, and future prospects of this field. It is anticipated that these insights will positively influence the advancement of stem cell applications in bone regeneration and offer new directions for researchers in this field.

## 2 Materials and methods

In our study, we conducted a comprehensive literature search on December 2, 2024, utilizing the Web of Science Core Collection as our primary data source. The search terms were as follows: topic = cell delivery OR cell implantation OR cell therapy AND topic = mesenchymal stem cells OR MSCs AND topic = bone regeneration OR osteogenesis AND publishing year = (January 1, 2013, to December 31, 2023). To assess the obtained literature, we employed standard bibliometric indicators commonly used in the scientific community, such as total citations, average citations, and the *H*-index as proposed by Hirsch (21). We obtained journal impact factors (IF) from Journal Citation Reports 2023 for analysis. We opted to use VOSviewer software to construct and visualize the bibliometric network of publications in our study (22). In our study’s visual depiction using VOSviewer, nodes represent various elements, with their sizes indicating the number of associated publications. The nodes’ colors signify the publication year, while the thickness of the interconnecting lines denotes the strength of collaboration or integration between these elements. CiteSpace (6.3. R1), developed by Professor Chaomei Chen, was used for country and institution collaboration analysis, journal dual-map overlay analysis, author collaboration and cited author analysis, cited literature and keyword cluster detection, and burst citation literature and keyword analysis (23). We conducted analyses using CiteSpace (6.3. R1), incorporating parameters such as the link retention factor (LRF = 2.5), the year of review (LBY = 5), e (*N* = 1), a time span from 2013 to 2023, 2 years per slice, link strength (cosine, within the slice range), selection criteria based on the *g*-index (*k* = 4), and minimum duration for keywords (MD = 2 as a reference).

## 3 Result

### 3.1 Global paper publication trend

**Figure 1:**
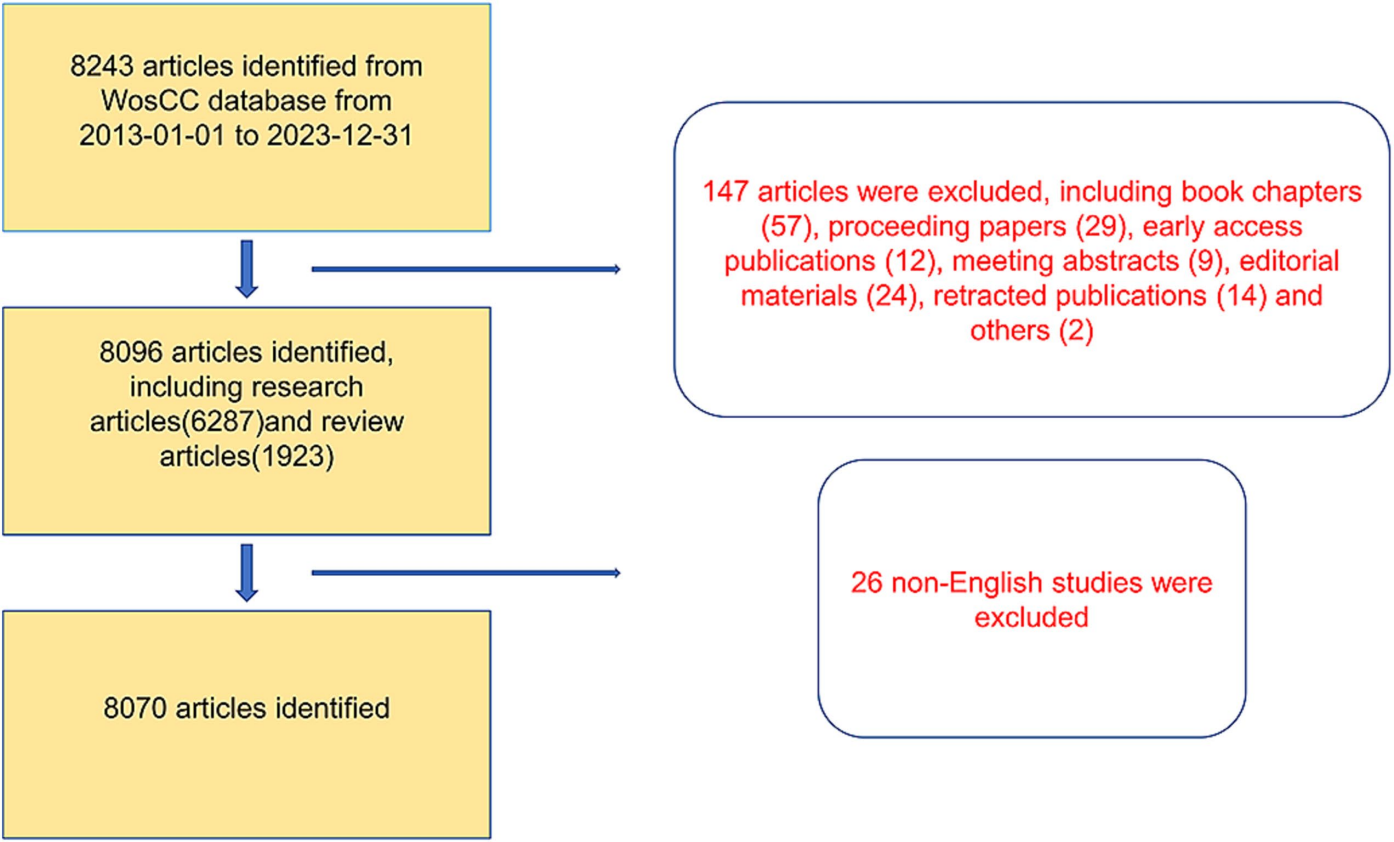
Flowchart of literature search and selection.

**Figure 2:**
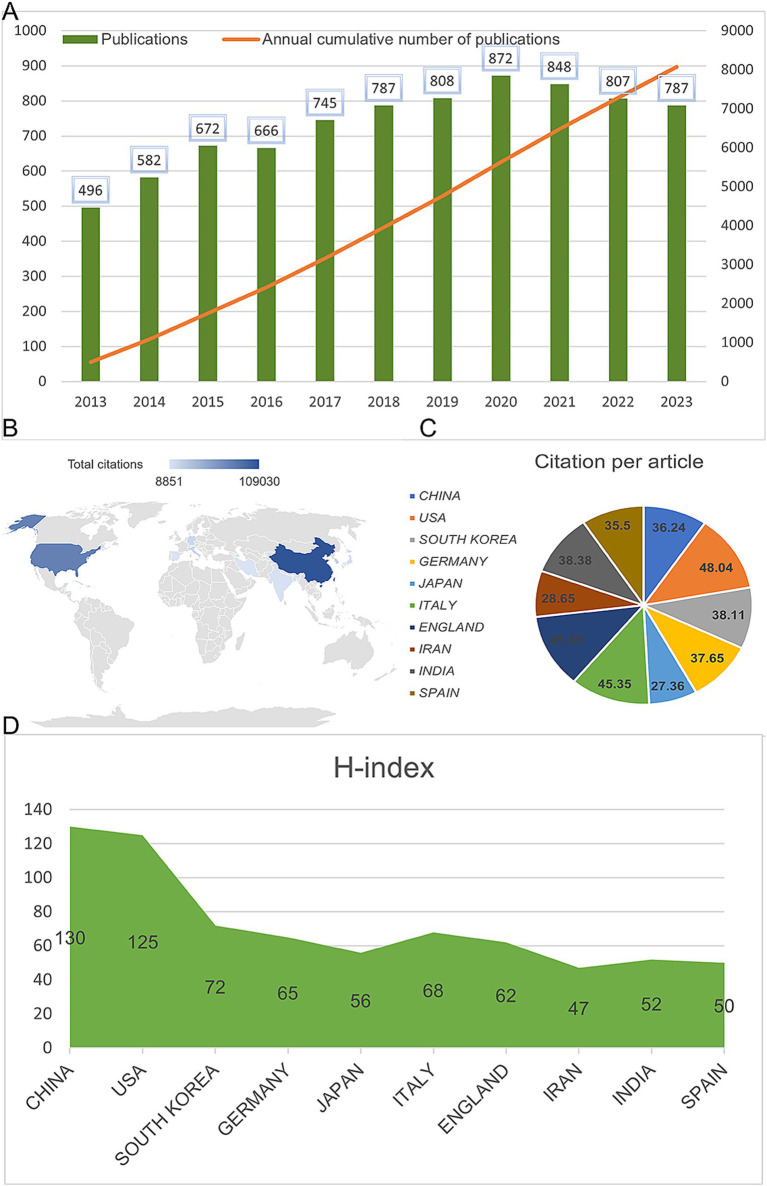
Global publication trends and citation frequency and *H*-index levels in the application of MSCs in bone regeneration in different countries/regions. **(A)** Annual publication volume and cumulative publication volume globally on mesenchymal stem cell therapy in bone regeneration. **(B)** Top 10 countries or regions in terms of total citations in the field. **(C)** Top 10 countries or regions in terms of average citations per paper in the field. **(D)** Top 10 countries and regions in terms of *H*-index in the field of mesenchymal stem cell therapy for bone regeneration.

A total of 8,243 articles were collected from the Web of Science database. Among them, book chapters (57 articles), proceeding papers (29 articles), early access publications (12 articles), meeting abstracts (9 articles), editorial materials (24 articles), retracted publications (14 articles) and others (2 articles) were excluded. Additionally, 26 non-English studies were excluded. Finally, 8,070 articles met the inclusion criteria for the Web of Science database (Figure 1). We summarized the global literature trends (Figure 2A). From 2013 to 2015, the annual publication counts steadily increased from 496 to 672. After 2016, the annual count fluctuated, peaking at 872 publications in 2020. Despite a slight decline in subsequent years, the overall trend in cumulative publications shows continuous growth, surpassing 8,000 by 2023. The top five countries with the highest number of articles are China (3,006 articles, 37.249%), the USA (1,668 articles, 20.669%), South Korea (545 articles, 6.753%), Germany (462 articles, 5.725%), and Japan (457 articles, 5.663%) (Table 1).

**Table 1:** The top 10 countries with the most publications related to MSCs therapy for bone regeneration.

Rank	Countries	Record count	Percentage (*N*/8,070)	Total citations	Citation per article	*H*-index
1	People’s Republic of China	3,006	37.249	109,030	36.24	130
2	USA	1,668	20.669	77,680	48.04	125
3	South Korea	545	6.753	20,769	38.11	72
4	Germany	462	5.725	17,393	37.65	65
5	Japan	457	5.663	12,504	27.36	56
6	Italy	385	4.771	17,461	45.35	68
7	England	314	3.891	14,397	45.85	62
8	Iran	314	3.891	8,997	28.65	47
9	India	268	3.321	10,286	38.38	52
10	Spain	249	3.086	8,851	35.5	50

### 3.2 Author collaboration and co-citation

**Figure 3:**
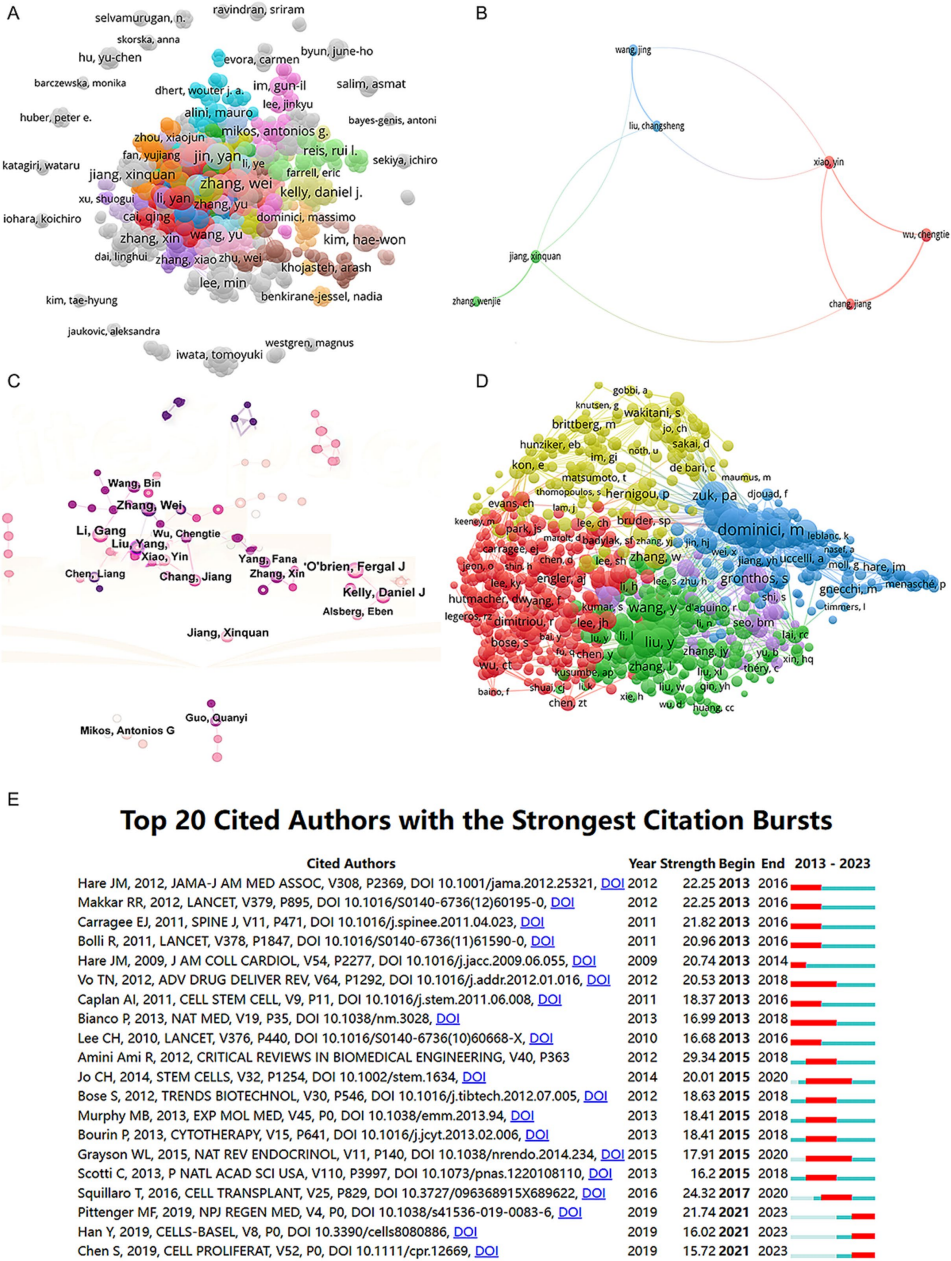
Mapping of authors in studies on MSCs therapy in bone regeneration. **(A)** Mapping of the identified authors in this field based on VOSviewer. The nodes represent countries/regions or institutions, and the lines connect them. The number of publications grows proportionally to the size of the nodes. The lines between the nodes represent the cooperation relationship, and the thickness of the connecting lines represents the strength of their cooperation; the closer the cooperation, the thicker the connecting lines. **(B)** Mapping of the seven-author co-authorship analysis in this field. **(C)** Author collaboration analysis based on CiteSpace. **(D)** Network visualization diagram of the co-cited authors of the publications. **(E)** Top 20 cited authors with the strongest citation bursts of publications related to MSCs therapy in bone regeneration. Author collaboration or co-cited authors are indicated by the node. The co-citation relationship is indicated by the line connecting the nodes. The node area grows as the number of co-citations increases. The colors represent different years. In **(C)**, the color changes from pink to purple from 2013 to 2023.

We collected a total of 8,070 articles involving 38,062 authors and visualized their collaboration networks (Figure 3A), emphasizing the co-authorship connections among the top seven authors (Figure 3B). The collaborative relationships between key authors were further analyzed using CiteSpace (Figure 3C). By examining the co-cited authors, we identified “Caplan A. I.,” “Dominici M.,” “Friedenstein A. J.,” “Liu Y.,” and “Pittenger M. F.” as the top five authors with the highest total connection strength, suggesting they may be central figures in the field (Figure 3D). Citation bursts, which indicate periods of frequent citations, revealed that these authors have experienced significant attention over time, serving as an important metric for their impact. The top 20 most-cited authors demonstrated the strongest citation bursts in publications related to mesenchymal stem cell applications (Figure 3E). “Amini Ami R.” ranked first with a burst strength of 29.34, followed by “Hare J. M.” with a burst strength of 22.25. Notably, “Pittenger M. F.” had the longest duration of citation bursts, spanning over 8 years (2015–2023). Interestingly, our analysis of the top 10 authors with the most publications revealed that nine out of the 10 are based in China (Table 2). Additionally, we summarized the top 10 funding agencies supporting research in this field (Table 3).

**Table 2:** The top 10 authors with the most publications related to MSCs therapy for bone regeneration.

Rank	Author	Record Count	Percentage (*N*/8,070)	Country
1	Liu Y.	111	1.375	China
2	Wang Y.	104	1.289	China
3	Zhang Y.	90	1.115	China
4	Zhang X.	80	0.991	China
5	Li Y.	78	0.967	China
6	Li J.	69	0.855	USA
7	Wang J.	61	0.756	China
8	Zhang J.	61	0.756	China
9	Wang X.	60	0.743	China
10	Zhang L.	58	0.719	China

**Table 3:** The top 10 funding agencies with the most publications related to MSCs therapy for bone regeneration.

Rank	Funding agencies	Record Count	Percentage (*N*/8,070)	Country
1	National Natural Science Foundation of China NSFC	2,011	24.919	China
2	United States Department of Health Human Services	692	8.575	USA
3	National Institutes of Health NIH USA	691	8.563	USA
4	Ministry of Education Culture Sports Science and Technology Japan MEXT	273	3.383	Japan
5	National Key Research Development Program of China	270	3.346	China
6	Japan Society for the Promotion of Science	261	3.234	Japan
7	European Union EU	243	3.011	European Union EU
8	Grants in Aid for Scientific Research KAKENHI	242	2.999	Japan
9	Fundamental Research Funds for the Central Universities	170	2.107	China
10	China Postdoctoral Science Foundation	160	1.983	China

### 3.3 National and institutional cooperation

**Figure 4:**
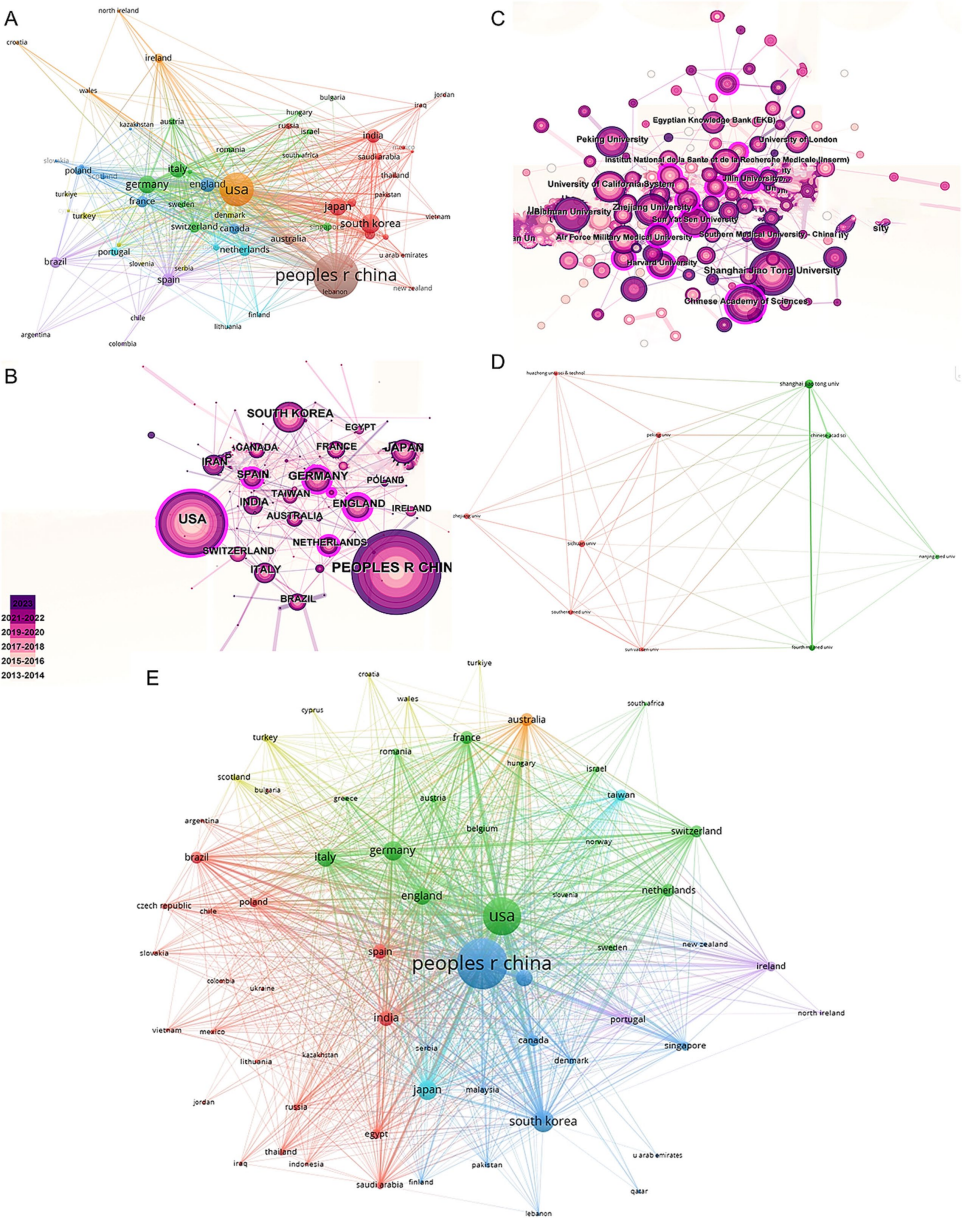
Mapping of countries/regions and institutions associated with MSCs therapy in bone regeneration. Country/regional collaboration analysis based on VOSviewer **(A)** and CiteSpace **(B)**. **(C)** Institutional collaboration analysis based on CiteSpace. **(D)** Mapping of the 10-institution co-authorship analysis on MSCs therapy in bone regeneration based on VOSviewer. **(E)** The bibliographic coupling of different countries of citations related to MSCs therapy in bone regeneration on VOSviewer.

In the co-authorship countries visualization shown in Figure 4A, it can be observed that the United States (strength = 579) has the highest total connection strength, followed by China (strength = 443), Germany (strength = 175), and Japan (strength = 121). In terms of publication output, China leads with 3,006 articles, followed by the United States (1,668 articles), South Korea (545 articles), Germany (462 articles), and Japan (457 articles) (Table 1). There is a relatively close collaboration among China, the USA, South Korea, Germany, and Japan (Figure 4B). Table 4 lists the top 10 institutions publishing the most related literature, with Shanghai Jiao Tong University ranked first, followed by Sichuan University and the Chinese Academy of Sciences. These results are also reflected in Figure 4C. Among these institutions, there are relatively close connections between the Chinese Academy of Sciences, Shanghai Jiao Tong University, Sun Yat-sen University, Southern Medical University, and Sichuan University (Figure 4D).

**Table 4:** The top 10 institutions with the most publications related to MSCs therapy for bone regeneration.

Rank	Institution	Article counts	Percentage (*N*/8,070)	Country	Total citations	Average citation	*H*-index
1	Shanghai Jiao Tong University	317	3.928	China	15,159	47.82	66
2	Sichuan University	231	2.862	China	6,982	30.23	48
3	Chinese Academy of Sciences	230	2.85	China	12,511	54.4	64
4	Peking University	166	2.057	China	7,108	42.82	46
5	Zhejiang University	160	1.983	China	6,506	40.66	47
6	University of California System	158	1.958	USA	8,306	52.57	50
7	Air Force Military Medical University	141	1.747	China	6,685	47.41	49
8	Southern Medical University China	135	1.673	China	4,358	32.28	37
9	Sun Yat-sen University	135	1.673	China	5,503	40.76	44
10	Harvard University	121	1.499	USA	7,643	63.17	48

### 3.4 Journals and research field

In this study, we identified 10 key research fields related to the topic (Table 5). Among these, Cell Biology had the highest number of publications (2,273 papers, *H*-index = 111), followed by Materials Science (2,197 papers, *H*-index = 126) and Engineering (1,762 papers, *H*-index = 108). The top 10 journals by publication volume were also identified, with Stem Cell Research and Therapy leading with 237 articles (impact factor = 7.1, 2023), followed by the International Journal of Molecular Sciences with 228 articles (impact factor = 4.9, 2023), and Acta Biomaterialia with 195 articles (impact factor = 9.4, 2023). Other notable journals included Biomaterials (192 articles, impact factor = 12.8, 2023) and Stem Cells International (162 articles, impact factor = 3.8, 2023). Interestingly, the publication numbers across these journals were relatively similar (Table 6).

**Table 5:** The top 10 research areas with the most publications related to MSCs therapy for bone regeneration.

Rank	Research areas	Article counts	Percentage (*N*/8,070)	Citation per article	*H*-index
1	Cell Biology	2,273	28.166	34.55	111
2	Materials Science	2,197	27.224	43.98	126
3	Engineering	1,762	21.834	38.05	108
4	Research Experimental Medicine	1,049	12.999	37.23	88
5	Science Technology Other Topics	885	10.967	46.49	96
6	Chemistry	785	9.727	42.51	86
7	Biotechnology Applied Microbiology	718	8.897	34.02	73
8	Biochemistry Molecular Biology	717	8.885	35.98	75
9	Pharmacology Pharmacy	547	6.778	35.01	66
10	Orthopedics	375	4.647	33.62	53

**Table 6:** The top 10 journals with the most publications related to MSCs therapy for bone regeneration.

Rank	Publication titles	Record count	Percentage (*N*/8,070)	Citation per article	*H*-index	IF
1	Stem Cell Research Therapy	237	2.937	48.76	55	7.1
2	International Journal of Molecular Sciences	228	2.825	32.71	48	4.9
3	Acta Biomaterialia	195	2.416	61.99	66	9.4
4	Biomaterials	192	2.379	78.88	78	12.8
5	Stem Cells International	162	2.007	36.95	40	3.8
6	Tissue Engineering Part A	148	1.834	29.56	39	3.5
7	Scientific Reports	128	1.586	35.77	40	3.8
8	Frontiers in Bioengineering and Biotechnology	121	1.499	19.26	25	4.3
9	Journal of Materials Chemistry B	118	1.462	36.27	39	6.1
10	Journal of Tissue Engineering and Regenerative Medicine	114	1.413	28.06	32	3.1

**Figure 5:**
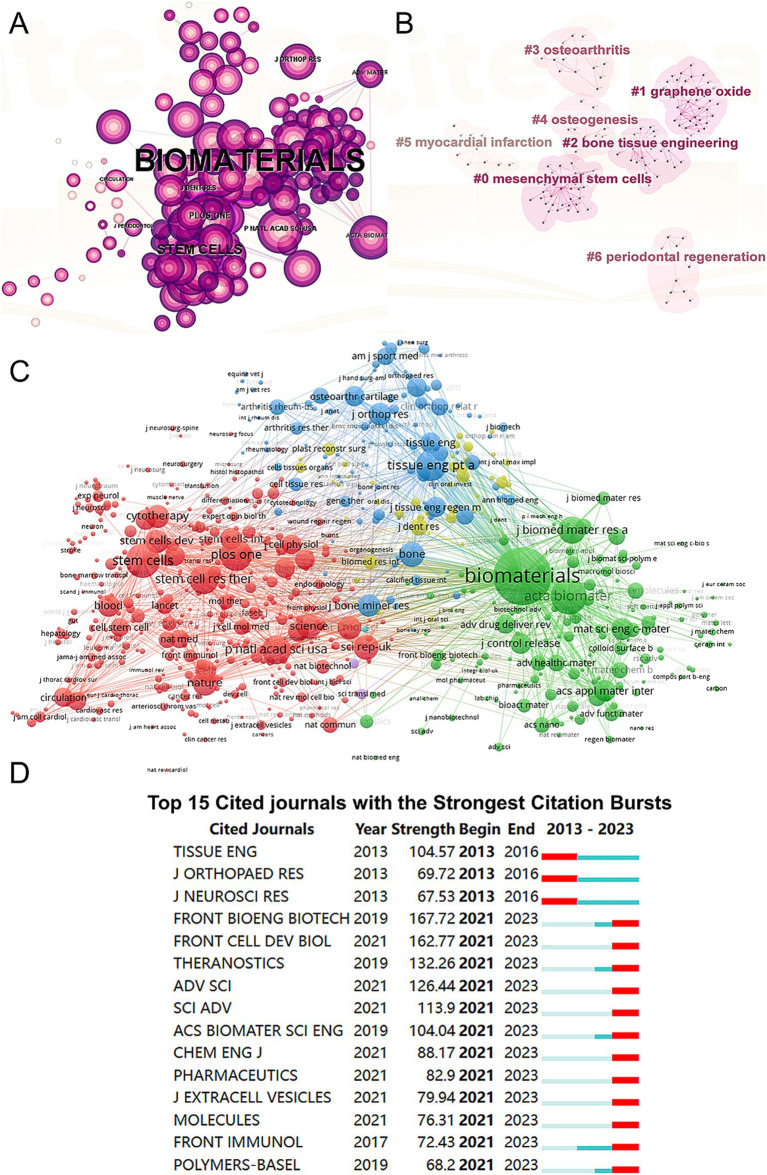
Articles published and cited in different journals on MSCs therapy in bone regeneration. **(A)** Visual analysis of the references cited across various journals using CiteSpace. **(B)** Clustering analysis of the co-cited journal network. **(C)** Mapping of the identified journals based on VOSviewer. **(D)** Top 15 journals with the strongest citation bursts of publications related to MSCs therapy in bone regeneration.

We conducted a visual analysis of reference citations across journals (Figure 5A) and performed co-clustering analysis using CiteSpace (Figure 5B). Key research hotspots identified include “mesenchymal stem cells,” “graphene oxide,” and “bone tissue engineering.” Using VOSviewer, we visualized the citation relationships among journals (Figure 5C), with the top five journals by total link strength being Biomaterials (2,717,148), Acta Biomaterialia (1,173,617), Stem Cells (895,597), PLoS One (841,759), and Tissue Engineering Part A (807,800). Additionally, we highlighted the top 15 journals with the highest citation rates in publications related to mesenchymal stem cell therapy for bone regeneration (Figure 5D).

### 3.5 Literature citation

Figure 4E shows the citation frequency by different countries and regions, with China leading at 2,432,564 citations, significantly ahead of the USA (2,344,484 citations), South Korea (661,042 citations), Italy (678,669 citations), and Germany (838,267 citations). Among the top 10 countries and regions with the highest average citation frequency (Table 1) (Figure 2B, 2C), the USA has the highest average citation frequency (48.04 citations), followed by England (45.85 citations), Italy (45.35 citations), India (38.38 citations), and South Korea (38.11 citations). We also analyzed the top 10 countries with the highest *H*-index in related publications (Figure 2D), with China (*H*-index = 130) and the USA (*H*-index = 125) leading, followed by South Korea (*H*-index = 72), Italy (*H*-index = 68), and Germany (*H*-index = 65).

### 3.6 Keyword

**Figure 6:**
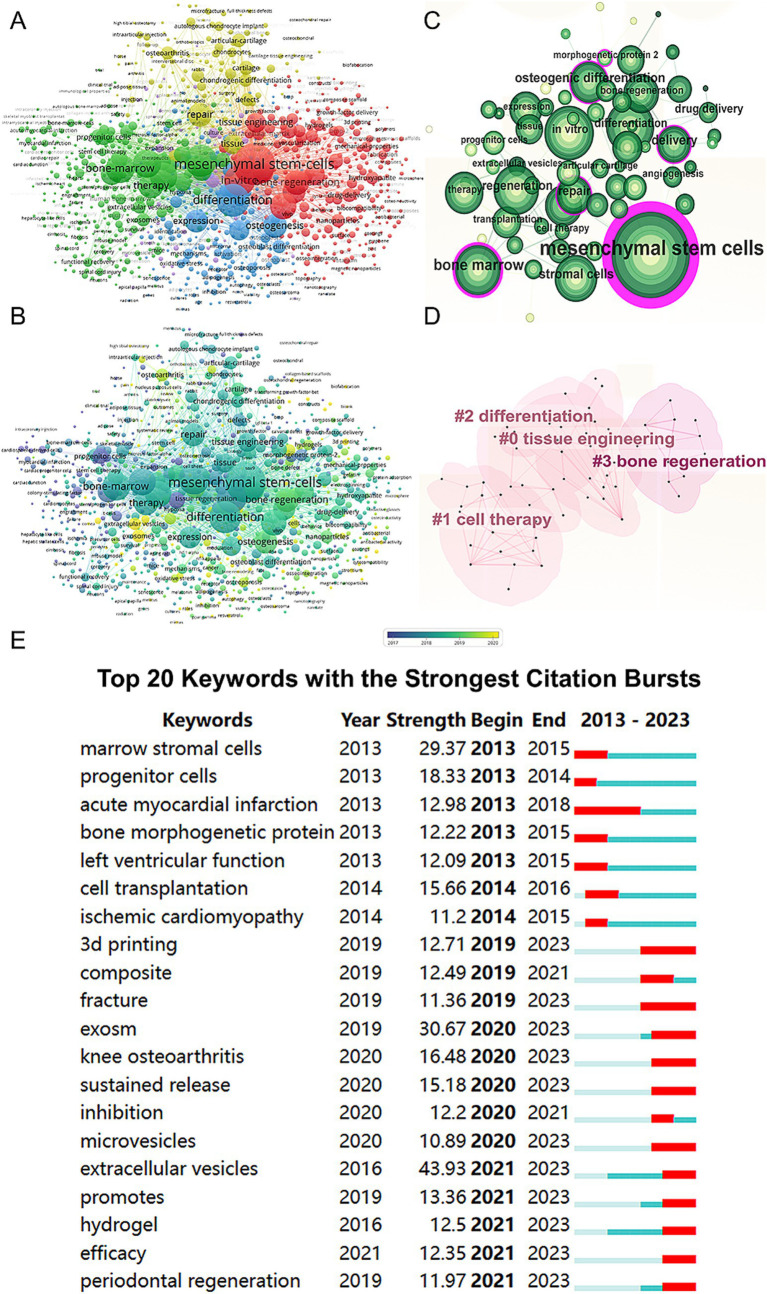
Mapping of keywords in studies on MSCs therapy in bone regeneration. **(A)** Network visualization of keywords by VOSviewer. **(B)** Distribution of keywords according to average publication year (blue: earlier, yellow: later) by VOSviewer. **(C)** Clustering analysis of the keyword network based on CiteSpace. **(D)** Keyword timeline visualization from 2013 to 2023 by CiteSpace. **(E)** The 20 keywords with the strongest citation bursts related to MSCs therapy in bone regeneration.

We conducted a keyword network visualization of the collected articles (Figure 6A). Among the 19,426 keywords, the top five with the highest total connection strength are “mesenchymal stem cells” (total link strength = 24,809), “differentiation” (total link strength = 16,154), “regeneration” (total link strength = 15,189), “*in vitro*” (total link strength = 14,407), and “bone-marrow” (total link strength = 12,233). We also visualized these keywords based on their average publication year (Figure 6B). Using CiteSpace, we created a visualization of these keywords (Figure 6C) and performed cluster analysis to establish a visual clustering of keywords (Figure 6D). Finally, we identified the 20 keywords with the most significant citation growth, finding that “extracellular vesicles” had the most significant citation growth strength (strength = 43.93, 2021–2023) (Figure 6E).

### References

3.7

**Figure 7:**
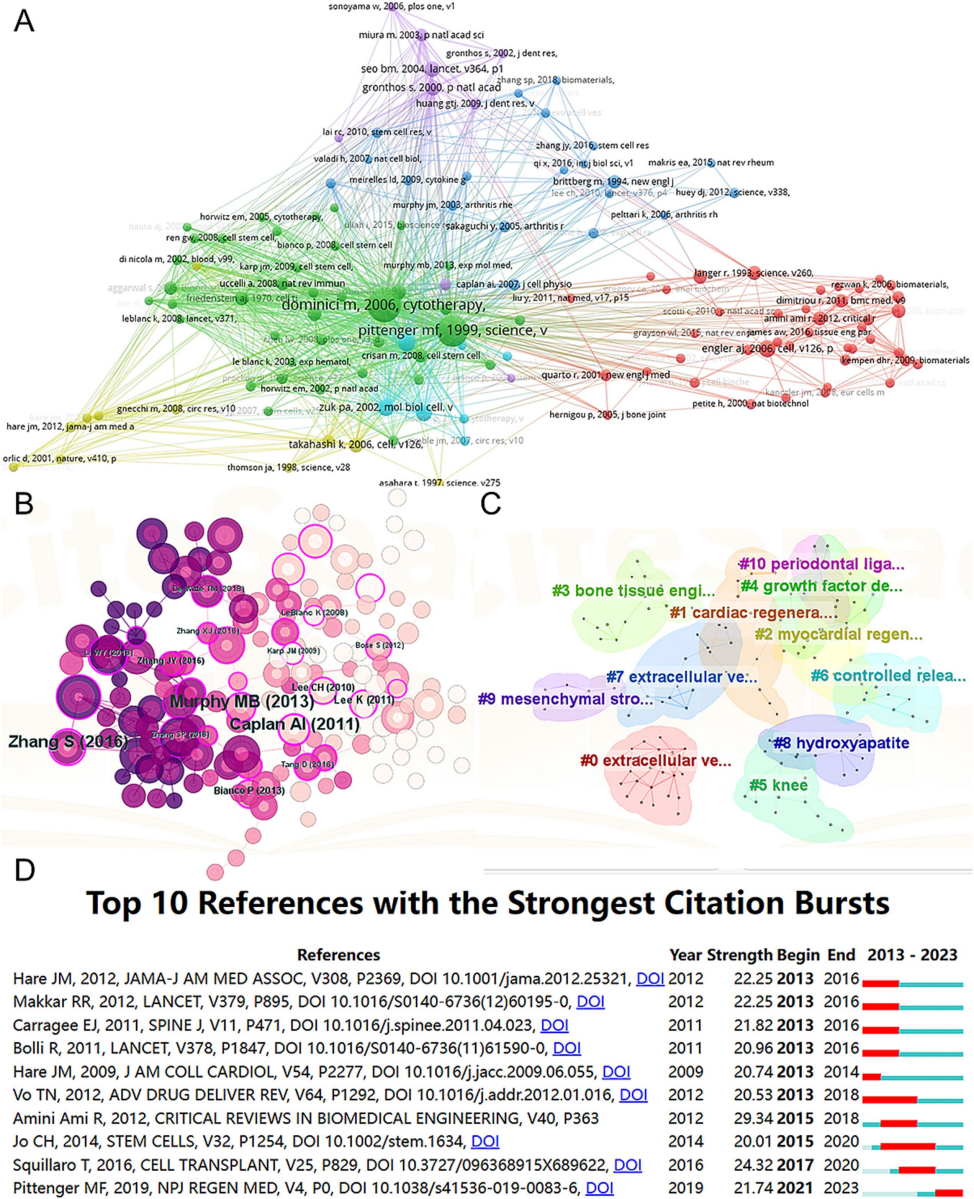
Mapping of cited references in studies on MSCs therapy in bone regeneration. Mapping of the co-cited references related to this field based on VOSviewer **(A)** and CiteSpace **(B)**. **(C)** Clustering analysis of the co-cited reference network based on CiteSpace. **(D)** Top 10 references with the strongest citation bursts of publications related to MSCs targeted therapy in bone regeneration.

A total of 128 out of the 261,144 cited references meet the threshold of being cited at least 80 times (Figure 7A). Among the top 5 most cited review articles (Table 7), “Clinical trials with mesenchymal stem cells: an update” was cited 1,023 times (24), followed by “Materials design for bone-tissue engineering” cited 1,016 times (25) and “Alginate-based biomaterials for regenerative medicine applications” cited 907 times (26). In the top 5 most cited research articles (Table 8), “Bone substitutes in orthopaedic surgery: from basic science to clinical practice” was cited 772 times (27), “Intra-articular injection of mesenchymal stem cells for the treatment of osteoarthritis of the knee: a proof-of-concept clinical trial” was cited 651 times (28) and “Extracellular vesicles improve post-stroke neuroregeneration and prevent postischemic immunosuppression” was cited 577 times (29). We performed co-cited references visualization for this field using CiteSpace (Figure 7B) and conducted a cluster visualization analysis of the references (Figure 7C) and found that “extracellular vesicles,” “cardiac regeneration,” and “bone tissue engineering” are hot topics in the references.

**Table 7:** The top five review articles with the most citations related to MSCs therapy for bone regeneration.

Rank	Title	Corresponding author	Journal	IF	Publication year	Total citations
1	Clinical trials with mesenchymal stem cells: an update	Squillaro, Tiziana	Cell Transplantation	3.2	2016	1,023
2	Materials design for bone-tissue engineering	Koons, Gerry L.	Nature Reviews Materials	79.8	2020	1,016
3	Alginate-based biomaterials for regenerative medicine applications	Sun, Jinchen	Materials	3.1	2013	907
4	Mesenchymal stem cells: environmentally responsive therapeutics for regenerative medicine	Murphy, Matthew B.	Experimental and Molecular Medicine	9.5	2013	869
5	Biomimetic porous scaffolds for bone tissue engineering	Wu, Shuilin	Materials Science & Engineering R-Reports	31.6	2014	848

**Table 8:** The top five research articles with the most citations related to MSCs therapy for bone regeneration.

Rank	Title	Corresponding author	Journal	IF	Publication year	Total citations
1	Bone substitutes in orthopaedic surgery: from basic science to clinical practice		Journal of Materials Science-Materials In Medicine	4.2	2014	772
2	Intra-articular injection of mesenchymal stem cells for the treatment of osteoarthritis of the knee: a proof-of-concept clinical trial	Jo, Chris Hyunchul	Stem Cells	4	2014	651
3	Extracellular vesicles improve post-stroke neuroregeneration and prevent postischemic immunosuppression	Doeppner, Thorsten R.	Stem Cells Translational Medicine	5.4	2015	577
4	Adipocyte accumulation in the bone marrow during obesity and aging impairs stem cell-based hematopoietic and bone regeneration	Ambrosi, Thomas H.	Cell Stem Cell	19.8	2017	556
5	Exosomes derived from human embryonic mesenchymal stem cells promote osteochondral regeneration	Zhang, S.	Osteoarthritis and Cartilage	7.2	2016	485

## 4 Discussion

### 4.1 Publication trends of this research

Our team conducted a bibliometric analysis of papers published between 2013 and 2023 to explore the progress and future directions in the field of mesenchymal stem cells for bone regeneration. During this period, the global number of papers on this topic showed a fluctuating upward trend, with a peak of 872 papers published in 2020. Although the number of papers published annually has generally increased, this trend was not statistically significant. On a global scale, China and the United States published far more papers than other countries/regions, together accounting for more than 50% of the total papers published in this field worldwide. Interestingly, we found that nine out of the top 10 authors with the highest citation counts were all from China. Furthermore, not only did China publish more papers than the U.S., but its *H*-index also surpassed that of the U.S., reflecting the significant contributions made by Chinese researchers in this field. However, in terms of average citation count, China ranked 7th, indicating that while China has a large number of papers, the academic impact of each paper is relatively lower. It is also worth noting that only four Chinese institutions appeared in the top 10 institutions by publication volume. This suggests that to enhance a country’s academic standing, it is not only necessary to establish top-tier research institutions and increase research investment but also to focus on improving research quality and avoiding an overemphasis on quantity.

Among the top 10 journals in this field, the leading ones focus on Cell Biology, Materials Science, Engineering, and Research Experimental Medicine. “Cell Biology” and “Materials Science” not only publish a large number of articles but also have high *H*-indices. This indicates that in this field, authors are more inclined to focus on cell biology and materials, which are highly relevant to the topic.

### 4.2 Hotpots and frontiers of this research

Highly explosive keywords can predict emerging directions. The current network reflects all keywords included in publication titles or abstracts, which we divide into two parts: mesenchymal stem cells and bone regeneration by tissue engineering.

#### 4.2.1 Mesenchymal stem cells

Mesenchymal stem cells (MSCs) are multipotent stem cells with unique self-renewal ability, pluripotency, and genomic stability (30–34). They are capable of exhibiting multipotent differentiation, making them promising candidates for cell therapy. MSCs are found not only in fetal tissues but also in many adult tissues, with few exceptions (35). According to the standards set by the International Society for Cell Therapy, the expression of specific cell surface markers is one of the fundamental characteristics of MSCs. Cells expressing CD73, CD90, and CD105 are considered MSCs with positive expression, while those expressing CD14, CD34, CD45, and HLA-DR are considered negative (36). MSCs can be induced to differentiate into adipocytes, chondrocytes, or osteocytes (37). In addition to their differentiation potential, MSCs also exhibit immunomodulatory properties, regulating, immune responses through the secretion of anti-inflammatory cytokines and interactions with immune cells (38–43). They also promote tissue repair and regeneration by secreting trophic factors that facilitate angiogenesis, inhibit cell apoptosis, and modulate the local microenvironment (44–48). Therefore, MSCs have a promising application in the field of bone regeneration and tissue engineering.

#### 4.2.2 Bone regeneration by tissue engineering

Bone regeneration is a highly intricate biological process that involves the regulation of inflammation by immune cells (49), the impact of neurotrophic factors on bone repair, angiogenesis providing nutrients, and the involvement of osteoblasts and mesenchymal stem cells in the formation and remodeling of new bone tissue (50, 51). These processes are finely regulated by a range of biological factors and signaling molecules, including growth factors, cytokines, and neurotrophic factors, whose coordinated actions ultimately determine the speed and quality of bone regeneration (52).

To enhance the regeneration of damaged or deficient bone, several strategies are available, including autologous bone grafts, vascularized fiber grafts, allogeneic grafts, bone tissue engineering, and distraction osteogenesis (53–56). Among these, bone tissue engineering, which combines cells, scaffold materials, and growth factors to repair, replace, or enhance tissue function, has emerged as a highly promising approach for treating bone defects (57, 58). In the field, biomaterials play a crucial role as they can serve as carriers for cells, allowing for targeted implantation at the lesion site, and providing an optimal growth environment for the implanted cells (59, 60).

Collectively, these approaches promote bone repair and regeneration by providing scaffold support, promoting cell proliferation and differentiation, stimulating angiogenesis, repairing and remodeling bone tissue, and ensuring graft integration and stability. These are widely utilized in the field of bone defects.

### 4.3 Prospects of this research

Based on the above analysis and illustrations, we can observe that over the past 10 years, mesenchymal stem cell (MSC) therapy in the field of bone regeneration has mainly gone through a process from basic research to applied research, and then to clinical application. Early research by scientists was primarily focused on the basic characteristics and differentiation mechanisms of cells, which laid a solid foundation for future studies. During the mid-term phase, the focus shifted towards exploring methods to optimize differentiation and proliferation conditions. With the continuous advancement of technology, researchers have increasingly concentrated on clinical applications and personalized treatment strategies. For instance, they have utilized various technologies to enhance the targeting of MSCs and have developed personalized stem cell treatments tailored to the individual, aiming to improve therapeutic outcomes.

Despite some progress, several challenges remain in using MSCs for bone regeneration therapy. Firstly, issues related to cellular aging and functional decline during *in vitro* expansion affect not only MSCs derived from patients but also allogeneic MSCs from healthy donors (61). Secondly, the immune response is another critical issue, as the efficacy of MSC transplantation is significantly influenced by the patient’s immune status, and allogeneic MSC transplantation may trigger immune rejection (62). Additionally, although technical advancements have improved cell viability, the post-implantation survival rate remains a pressing issue.

To overcome these challenges, future research needs to further explore and develop new strategies and methods. This includes improving *in vitro* expansion techniques to minimize cellular aging, optimizing immunomodulatory strategies to reduce the risk of immune rejection, and enhancing the survival rate and functionality of MSCs *in vivo*. Through these efforts, MSC targeted therapies are expected to achieve safer and more effective applications in the field of bone regeneration.

### 4.4 Advances and limitations of this research

This study employed bibliometric and visualization analysis methods to explore the literature on mesenchymal stem cell therapy for bone regeneration over the past 10 years. While our findings are comprehensive and objective, there are inevitable limitations. Firstly, we used only the Web of Science Core Collection (WOSCC) for literature retrieval, excluding other databases such as PubMed, Scopus, Cochrane, and Embase. Although WOSCC is a widely used authoritative comprehensive database, this may have led to the omission of some relevant literature, resulting in potential selection bias. Secondly, we excluded non-English literature and non-research/review articles, which may overlook relevant studies published in other languages. Chinese publications, in particular, have made significant contributions in this field. Review and research articles are valuable publication types, each with its unique role and value. Therefore, we did not separately discuss research and review articles. Furthermore, we did not include articles published after January 2024, which may introduce a degree of predictive bias in the relevance analysis. Lastly, we did not consider the quality of the publications in certain analyses, treating high-quality and low-quality publications equally.

## 5 Conclusion

We conducted an in-depth study on the application of MSCs in bone regeneration therapy, summarizing the development trends in this field over the past 10 years through comprehensive literature analysis and visualization methods. We systematically analyzed global research dynamics and identified influential authors, institutions, and journals. Through co-occurrence analysis of keywords and research directions, we accurately captured the hotspots and emerging trends of MSCs in bone regeneration therapy. Our study comprehensively summarized the current status of MSCs in bone regeneration therapy, outlined the main focuses of research, and provided a forward-looking analysis of future trends. Our work aims to deepen the understanding of MSCs in bone regeneration therapy, provide insights to researchers, guide future research directions, and promote the translation and application of research outcomes. In the future, we will continue to explore the potential applications and mechanisms of MSCs, optimize treatment regimens, and improve treatment efficacy and biocompatibility. Additionally, we will enhance interdisciplinary collaboration to advance the clinical application of MSCs in bone regeneration therapy, aiming to provide more effective treatment options for patients and improve their quality of life. In summary, our study comprehensively elucidates the current status of MSCs in the field of bone regeneration therapy and provides an outlook on future directions, aiming to drive progress in this field, accelerate the translation of relevant research outcomes into clinical practice, and contribute to the development of bone regeneration therapy.

## References

[1] Knight MN Hankenson KD. Mesenchymal stem cells in bone regeneration. Adv Wound Care. (2013) 2:306–16. doi: 10.1089/wound.2012.0420, PMID: 24527352 PMC3842877

[2] Marzona L Pavolini B. Play and players in bone fracture healing match. Clin Cases Miner Bone Metab. (2009) 6:159–62. PMID: 22461167 PMC2781220

[3] Aspenberg P Sandberg O. Distal radial fractures heal by direct woven bone formation. Acta Orthop. (2013) 84:297–300. doi: 10.3109/17453674.2013.792769, PMID: 23570338 PMC3715812

[4] Uhthoff HK Rahn BA. Healing patterns of metaphyseal fractures. Clin Orthop Relat Res. (1981) 160, 295–303. PMID: 7285432

[5] Loi F Córdova LA Pajarinen J Lin TH Yao Z Goodman SB. Inflammation, fracture and bone repair. Bone. (2016) 86:119–30. doi: 10.1016/j.bone.2016.02.020, PMID: 26946132 PMC4833637

[6] Friedenstein AJ Piatetzky-Shapiro II Petrakova KV. Osteogenesis in transplants of bone marrow cells. J Embryol Exp Morphol. (1966) 16:381–90. PMID: 5336210

[7] Dominici M Le Blanc K Mueller I Slaper-Cortenbach I Marini F Krause D. Minimal criteria for defining multipotent mesenchymal stromal cells. The International Society for Cellular Therapy position statement. Cytotherapy. (2006) 8:315–7. doi: 10.1080/14653240600855905, PMID: 16923606

[8] Uccelli A Moretta L Pistoia V. Mesenchymal stem cells in health and disease. Nat Rev Immunol. (2008) 8:726–36. doi: 10.1038/nri2395 19172693

[9] Friedenstein AJ Chailakhyan RK Latsinik NV Panasyuk AF Keiliss-Borok IV. Stromal cells responsible for transferring the microenvironment of the hemopoietic tissues. Cloning *in vitro* and retransplantation *in vivo*. Transplantation. (1974) 17:331–40. doi: 10.1097/00007890-197404000-00001, PMID: 4150881

[10] Fridenshteĭn A Piatetzky-Shapiro II Petrakova KV. Osteogenesis in transplants of bone marrow cells. Arkh Anat Gistol Embriol. (1969) 56:3–11. 4903779

[11] Zhang B Yang L Zeng Z Feng Y Wang X Wu X. Leptin potentiates BMP9-induced osteogenic differentiation of mesenchymal stem cells through the activation of JAK/STAT signaling. Stem Cells Dev. (2020) 29:498–510. doi: 10.1089/scd.2019.0292, PMID: 32041483 PMC7153647

[12] Zhang S Tuk B van de Peppel J Kremers GJ Koedam M Pesch GR. Microfluidic evidence of synergistic effects between mesenchymal stromal cell-derived biochemical factors and biomechanical forces to control endothelial cell function. Acta Biomater. (2022) 151:346–59. doi: 10.1016/j.actbio.2022.08.025, PMID: 35995408

[13] Roche ET Hastings CL Lewin SA Shvartsman D Brudno Y Vasilyev NV. Comparison of biomaterial delivery vehicles for improving acute retention of stem cells in the infarcted heart. Biomaterials. (2014) 35:6850–8. doi: 10.1016/j.biomaterials.2014.04.114, PMID: 24862441 PMC4051834

[14] Jokerst JV Khademi C Gambhir SS. Intracellular aggregation of multimodal silica nanoparticles for ultrasound-guided stem cell implantation. Sci Transl Med. (2013) 5:177ra35. doi: 10.1126/scitranslmed.3005228 PMC3839309 23515077

[15] Wong JKU Mehta A Vũ TT Yeo GC. Cellular modifications and biomaterial design to improve mesenchymal stem cell transplantation. Biomater Sci. (2023) 11:4752–73. doi: 10.1039/D3BM00376K, PMID: 37233031

[16] Yao ZC Yang YH Kong J Zhu Y Li L Chang C. Biostimulatory micro-fragmented nanofiber-hydrogel composite improves mesenchymal stem cell delivery and soft tissue remodeling. Small. (2022) 18:e2202309. doi: 10.1002/smll.202202309 35948487 PMC9994419

[17] He L Zhou Q Zhang H Zhao N Liao L. PF127 hydrogel-based delivery of exosomal CTNNB1 from mesenchymal stem cells induces osteogenic differentiation during the repair of alveolar bone defects. Nanomaterials. (2023) 13:1083. doi: 10.3390/nano13061083 36985977 PMC10058633

[18] Teo JY Ko E Leong J Hong J Jeon JS Yang YY. Surface tethering of stromal cell-derived factor-1α carriers to stem cells enhances cell homing to ischemic muscle. Nanomedicine. (2020) 28:102215. doi: 10.1016/j.nano.2020.102215, PMID: 32438106 PMC7438260

[19] Chen W Li M Cheng H Yan Z Cao J Pan B. Overexpression of the mesenchymal stem cell Cxcr4 gene in irradiated mice increases the homing capacity of these cells. Cell Biochem Biophys. (2013) 67:1181–91. doi: 10.1007/s12013-013-9632-6, PMID: 23712865

[20] Krauskopf E. A bibiliometric analysis of the Journal of Infection and Public Health: 2008–2016. J Infect Public Health. (2018) 11:224–9. doi: 10.1016/j.jiph.2017.12.011, PMID: 29361505

[21] Hirsch JE. An index to quantify an individual’s scientific research output. Proc Natl Acad Sci USA. (2005) 102:16569–72. doi: 10.1073/pnas.0507655102, PMID: 16275915 PMC1283832

[22] van Eck NJ Waltman L. Software survey: VOSviewer, a computer program for bibliometric mapping. Scientometrics. (2010) 84:523–38. doi: 10.1007/s11192-009-0146-3, PMID: 20585380 PMC2883932

[23] Synnestvedt MB Chen C Holmes JH. CiteSpace II: visualization and knowledge discovery in bibliographic databases. AMIA Annu Symp Proc. (2005) 2005:724–8. 16779135 PMC1560567

[24] Squillaro T Peluso G Galderisi U. Clinical trials with mesenchymal stem cells: an update. Cell Transplant. (2016) 25:829–48. doi: 10.3727/096368915X689622 26423725

[25] Koons GL Diba M Mikos AG. Materials design for bone-tissue engineering. Nat Rev Mater. (2020) 5:584–603. doi: 10.1038/s41578-020-0204-2

[26] Sun J Tan H. Alginate-based biomaterials for regenerative medicine applications. Materials. (2013) 6:1285–309. doi: 10.3390/ma6041285, PMID: 28809210 PMC5452316

[27] Campana V Milano G Pagano E Barba M Cicione C Salonna G. Bone substitutes in orthopaedic surgery: from basic science to clinical practice. J Mater Sci Mater Med. (2014) 25:2445–61. doi: 10.1007/s10856-014-5240-2, PMID: 24865980 PMC4169585

[28] Jo CH Lee YG Shin WH Kim H Chai JW Jeong EC. Intra-articular injection of mesenchymal stem cells for the treatment of osteoarthritis of the knee: a proof-of-concept clinical trial. Stem Cells. (2014) 32:1254–66. doi: 10.1002/stem.1634, PMID: 24449146

[29] Doeppner TR Herz J Gorgens A Schlechter J Ludwig AK Radtke S. Extracellular vesicles improve post-stroke neuroregeneration and prevent postischemic immunosuppression. Stem Cells Transl Med. (2015) 4:1131–43. doi: 10.5966/sctm.2015-0078, PMID: 26339036 PMC4572905

[30] Prockop DJ. Marrow stromal cells as stem cells for nonhematopoietic tissues. Science. (1997) 276:71–4. doi: 10.1126/science.276.5309.71 9082988

[31] Granero-Molto F Weis JA Longobardi L Spagnoli A. Role of mesenchymal stem cells in regenerative medicine: application to bone and cartilage repair. Expert Opin Biol Ther. (2008) 8:255–68. doi: 10.1517/14712598.8.3.255 18294098

[32] Salem HK Thiemermann C. Mesenchymal stromal cells: current understanding and clinical status. Stem Cells. (2010) 28:585–96. doi: 10.1002/stem.269, PMID: 19967788 PMC2962904

[33] Bianchi G Borgonovo G Pistoia V Raffaghello L. Immunosuppressive cells and tumour microenvironment: focus on mesenchymal stem cells and myeloid derived suppressor cells. Histol Histopathol. (2011) 26:941–51. doi: 10.14670/HH-26.941, PMID: 21630223

[34] Wei X Yang X Han ZP Qu FF Shao L Shi YF. Mesenchymal stem cells: a new trend for cell therapy. Acta Pharmacol Sin. (2013) 34:747–54. doi: 10.1038/aps.2013.50, PMID: 23736003 PMC4002895

[35] Ullah I Subbarao RB Rho GJ. Human mesenchymal stem cells—current trends and future prospective. Biosci Rep. (2015) 35:e00191. doi: 10.1042/BSR20150025, PMID: 25797907 PMC4413017

[36] Tuan RS. Stemming cartilage degeneration: adult mesenchymal stem cells as a cell source for articular cartilage tissue engineering. Arthritis Rheum. (2006) 54:3075–8. doi: 10.1002/art.22148, PMID: 17009225

[37] Spees JL Lee RH Gregory CA. Mechanisms of mesenchymal stem/stromal cell function. Stem Cell Res Ther. (2016) 7:125. doi: 10.1186/s13287-016-0363-7, PMID: 27581859 PMC5007684

[38] Spaggiari GM Capobianco A Becchetti S Mingari MC Moretta L. Mesenchymal stem cell-natural killer cell interactions: evidence that activated NK cells are capable of killing MSCs, whereas MSCs can inhibit IL-2-induced NK-cell proliferation. Blood. (2006) 107:1484–90. doi: 10.1182/blood-2005-07-2775, PMID: 16239427

[39] Chen FH Tuan RS. Mesenchymal stem cells in arthritic diseases. Arthritis Res Ther. (2008) 10:223. doi: 10.1186/ar2514, PMID: 18947375 PMC2592798

[40] English K Barry FP Mahon BP. Murine mesenchymal stem cells suppress dendritic cell migration, maturation and antigen presentation. Immunol Lett. (2008) 115:50–8. doi: 10.1016/j.imlet.2007.10.002, PMID: 18022251

[41] Kim J Hematti P. Mesenchymal stem cell-educated macrophages: a novel type of alternatively activated macrophages. Exp Hematol. (2009) 37:1445–53. doi: 10.1016/j.exphem.2009.09.004, PMID: 19772890 PMC2783735

[42] Németh K Leelahavanichkul A Yuen PS Mayer B Parmelee A Doi K. Bone marrow stromal cells attenuate sepsis via prostaglandin E_2_-dependent reprogramming of host macrophages to increase their interleukin-10 production. Nat Med. (2009) 15:42–9. doi: 10.1038/nm.1905, PMID: 19098906 PMC2706487

[43] English K. Mechanisms of mesenchymal stromal cell immunomodulation. Immunol Cell Biol. (2013) 91:19–26. doi: 10.1038/icb.2012.56 23090487

[44] Gnecchi M Zhang Z Ni A Dzau VJ. Paracrine mechanisms in adult stem cell signaling and therapy. Circ Res. (2008) 103:1204–19. doi: 10.1161/CIRCRESAHA.108.176826, PMID: 19028920 PMC2667788

[45] Ahmed LA Al-Massri KF. Directions for enhancement of the therapeutic efficacy of mesenchymal stem cells in different neurodegenerative and cardiovascular diseases: current status and future perspectives. Curr Stem Cell Res Ther. (2021) 16:858–76. doi: 10.2174/1574888X16666210303151237, PMID: 33655876

[46] Xu Z Wang B Huang R Guo M Han D Yin L. Efforts to promote osteogenesis-angiogenesis coupling for bone tissue engineering. Biomater Sci. (2024) 12:2801–30. doi: 10.1039/D3BM02017G, PMID: 38683241

[47] Mo X Zhang D Liu K Zhao X Li X Wang W. Nano-hydroxyapatite composite scaffolds loaded with bioactive factors and drugs for bone tissue engineering. Int J Mol Sci. (2023) 24:1291. doi: 10.3390/ijms24021291, PMID: 36674810 PMC9867487

[48] Monsel A Zhu YG Gudapati V Lim H Lee JW. Mesenchymal stem cell derived secretome and extracellular vesicles for acute lung injury and other inflammatory lung diseases. Expert Opin Biol Ther. (2016) 16:859–71. doi: 10.1517/14712598.2016.1170804, PMID: 27011289 PMC5280876

[49] Bai L Liu Y Du Z Weng Z Yao W Zhang X. Differential effect of hydroxyapatite nano-particle versus nano-rod decorated titanium micro-surface on osseointegration. Acta Biomater. (2018) 76:344–58. doi: 10.1016/j.actbio.2018.06.023, PMID: 29908975

[50] Bai L Chen P Zhao Y Hang R Yao X Tang B. A micro/nano-biomimetic coating on titanium orchestrates osteo/angio-genesis and osteoimmunomodulation for advanced osseointegration. Biomaterials. (2021) 278:121162. doi: 10.1016/j.biomaterials.2021.121162, PMID: 34628191

[51] Kusumbe AP Ramasamy SK Adams RH. Coupling of angiogenesis and osteogenesis by a specific vessel subtype in bone. Nature. (2014) 507:323–8. doi: 10.1038/nature13145, PMID: 24646994 PMC4943525

[52] Bai L Song P Su J. Bioactive elements manipulate bone regeneration. Biomater Transl. (2023) 4:248–69. doi: 10.12336/biomatertransl.2023.04.005, PMID: 38282709 PMC10817798

[53] Aronson J. Limb-lengthening, skeletal reconstruction, and bone transport with the Ilizarov method. J Bone Joint Surg Am. (1997) 79:1243–58. doi: 10.2106/00004623-199708000-00019, PMID: 9278087

[54] Green SA Jackson JM Wall DM Marinow H Ishkanian J. Management of segmental defects by the Ilizarov intercalary bone transport method. Clin Orthop Relat Res. (1992) 280, 136–42. PMID: 1611733

[55] Giannoudis PV Dinopoulos H Tsiridis E. Bone substitutes: an update. Injury. (2005) 36:S20–7. doi: 10.1016/j.injury.2005.07.029 16188545

[56] Giannoudis PV Einhorn TA. Bone morphogenetic proteins in musculoskeletal medicine. Injury. (2009) 40:S1–3. doi: 10.1016/S0020-1383(09)00642-1 20082783

[57] Chen X Wu T Bu Y Yan H Lin Q. Fabrication and biomedical application of alginate composite hydrogels in bone tissue engineering: a review. Int J Mol Sci. (2024) 25:7810. doi: 10.3390/ijms25147810 39063052 PMC11277200

[58] Mooney DJ Vandenburgh H. Cell delivery mechanisms for tissue repair. Cell Stem Cell. (2008) 2:205–13. doi: 10.1016/j.stem.2008.02.005 18371446

[59] Li X Lin H Yu Y Lu Y He B Liu M. In situ rapid-formation sprayable hydrogels for challenging tissue injury management. Adv Mater. (2024) 36:e2400310. doi: 10.1002/adma.202400310 38298099

[60] Wang S Liu J Zhou L Xu H Zhang D Zhang X. Research progresses on mitochondrial-targeted biomaterials for bone defect repair. Regen Biomater. (2024) 11:rbae082. doi: 10.1093/rb/rbae082, PMID: 39055307 PMC11272180

[61] Shuai Y Liao L Su X Yu Y Shao B Jing H. Melatonin treatment improves mesenchymal stem cells therapy by preserving stemness during long-term *in vitro* expansion. Theranostics. (2016) 6:1899–917. doi: 10.7150/thno.15412, PMID: 27570559 PMC4997245

[62] Sui BD Hu CH Zheng CX Shuai Y He XN Gao PP. Recipient glycemic micro-environments govern therapeutic effects of mesenchymal stem cell infusion on osteopenia. Theranostics. (2017) 7:1225–44. doi: 10.7150/thno.18181, PMID: 28435461 PMC5399589

